# The C55A Single Nucleotide Polymorphism in *CTLA-4* Gene, a New Possible Biomarker in Thyroid Autoimmune Pathology Such as Hashimoto’s Thyroiditis

**DOI:** 10.3390/diagnostics13152517

**Published:** 2023-07-28

**Authors:** Alin-Dan Chiorean, Mihaela Laura Vica, Ștefana Bâlici, Gheorghe Zsolt Nicula, Nicoleta Răcătăianu, Mădălina Adriana Bordea, Laura-Mihaela Simon, Horea Vladi Matei

**Affiliations:** 1 Department of Cell and Molecular Biology, Faculty of Medicine, “Iuliu Hațieganu” University of Medicine and Pharmacy, 400349 Cluj-Napoca, Romania; chiorean.alin@umfcluj.ro (A.-D.C.); sbalici@umfcluj.ro (Ș.B.); gnicula@umfcluj.ro (G.Z.N.); hmatei@umfcluj.ro (H.V.M.); 2 Clinical Laboratory, Emergency Clinical Hospital for Children, 400370 Cluj-Napoca, Romania; 3 Institute of Legal Medicine Cluj-Napoca, 400006 Cluj-Napoca, Romania; 4 Integrated Ambulatory of Endocrinology, Infectious Diseases Clinical Hospital, 400000 Cluj-Napoca, Romania; comanniko@yahoo.com; 5 Department of Microbiology, Faculty of Medicine, “Iuliu Hațieganu” University of Medicine and Pharmacy, 400349 Cluj-Napoca, Romania; bordea_madalina@yahoo.com (M.A.B.); lauramihaelasimon@yahoo.com (L.-M.S.)

**Keywords:** Hashimoto’s thyroiditis, *CTLA-4* gene, new biomarker, single nucleotide polymorphisms, thyroid autoimmune pathology, exon

## Abstract

Hashimoto’s thyroiditis (HT) is a chronic autoimmune disorder characterized by the production of autoantibodies against the thyroid gland. Different studies have shown that several genes may be associated with HT, which explains why patients often have family members with thyroiditis or other autoimmune diseases. The aim of this case-control study was to evaluate the correlation between polymorphisms at the level of exon 1 from the *CTLA-4* gene and the susceptibility to developing HT. In this study, we found that there is no statistically significant association between the polymorphism rs231775 (A22G in exon 1) of the *CTLA-4* gene and a genetic predisposition to HT. In contrast, a strong association was discovered for the first time between C55A in exon 1 of the *CTLA-4* gene and HT. Our findings suggest that there is a genetic relationship between the *CTLA-4* (+55A/C) genotype and the seropositivity against thyroid autoantigens, such as anti-thyroid peroxidase (ATPO) and anti-thyroglobulin antibodies (ATG).

## 1. Introduction

Hashimoto’s thyroiditis (HT) is a chronic autoimmune inflammation of the thyroid gland [1]. It was originally described over a century ago as lymphocyte infiltration, parenchymal atrophy, and fibrosis [2]. HT is the most common cause of hypothyroidism worldwide [3,4], but it is not completely defined from an etiopathogenetic point of view. Currently considered the most common autoimmune disease [5,6], HT is the most frequent endocrine disorder of the decade [7]. 

Many articles report the presence of a genetic predisposition in autoimmune thyroid diseases (AITDs) among heterogeneous populations [8,9,10]. AITDs, including HT and Graves’ disease (GD) [11,12], are related to environmental and genetic factors [13]. Several studies have investigated the association between different SNPs of the *CTLA-4* gene and AITDs, including HT and GD. Thus, the *CTLA-4* A49G and C318T genotypes seem to be susceptibility factors, while the C1822T and CT60 A/G genotypes do not influence the risk of AITDs [14,15,16,17]. 

Concordance was found in monozygotic twins and among populations with a family history of genetic diseases [18,19]. Consequently, family genetic testing is crucial in establishing an early diagnosis [9]. Moreover, segregation analysis tests highlighted a vertical transmission in terms of the production of autoantibodies that follow a dominant Mendelian pattern of genetic inheritance [20]. The prevalence of HT in females is another argument that supports the important role of genetic factors in the development of AITDs [1]. The fact that the healthy siblings of AITDs patients with positive autoantibodies demonstrates that the genetic locus responsible for seropositivity is different from the one involved in the control of the clinical manifestations of AITDs (fatigue, dry skin, constipation, increased sensitivity to cold, etc.) [21,22,23,24]. The locus of the gene involved in the synthesis of the *Cytotoxic T-Lymphocyte Antigen* (*CTLA-4*) is located on the long arm, band 33 of chromosome 2 [25,26]. The downregulated T cell activation can happen after the interaction between the B7 molecule with immunoregulatory function, the latter being encoded by the *CTLA-4* gene and expressed on the surface of activated T cells [27,28,29]. The Single Nucleotide Polymorphisms (SNPs) in the *CTLA-4* gene can also influence the T-effector activity [14,29,30,31]. It is thought to be responsible for controlling the production of anti-thyroid antibodies (Ac.) [32,33]. A study demonstrated that patients with CTLA-4 (22/G) A/G (rs231775) have high titers of anti-thyroglobulin (TG) antibodies [18]. Conversely, Single Nucleotide Polymorphisms (SNP) with GG/AG genotypes is associated with relevant titers of anti-thyroid peroxidase (TPO) and anti-TG antibodies. The production of anti-TG and anti-TPO thyroid autoantibodies is a specific feature of AITDs [1,10,33]. These autoantibodies are used as markers for the evaluation of various forms of AITDs, such as hypothyroidism, HT, and postpartum thyroiditis (PPT) [1,21,22,23,24,34]. Another study proved that anti-TPO and TG antibodies develop in HT as a result of follicular cells damages, produced by T lymphocytes mediated cytotoxicity [18,32]. PPT patients with positive anti TPO-antibodies are at high risk for developing permanent hypothyroidism [9,34,35]. In contrast, PPT patients with negative anti-TPO antibodies return to the status of euthyroid activity after a transient period of hypothyroidism [9,34,35,36].

Despite extensive investigations of CTLA-4 SNPs, the association between +22A/G rs231775 SNP and thyroid auto-antibody production in Asian patients has not been demonstrated [36,37,38,39,40,41]. 

Therefore, in the present study, we aimed to determine whether there is a role played by *CTLA-4* genotypes at rs231775 and other new SNPs in exon 1, which we determined using Sanger sequencing among seropositive patients to evaluate their role between *CTLA-4* genotypes and susceptibility and influence in the increase of anti-TPO and anti-TG antibody titers in AITDs.

We demonstrate for the first time that C55A polymorphism is positively associated with disease, specifically HT.

## 2. Materials and Methods

### 2.1. Study Approval for the Collection and Analysis of Biological Samples from the Subjects

This study is a case-control type, carried out in the period between 2019 and 2021 in the northwest of Transylvania (Romania). The present study had the approval of the ethics commissions of “Iuliu Hațieganu” University of Medicine and Pharmacy Cluj-Napoca and Clinical Hospital of Infectious Diseases Cluj-Napoca (269/30 July 2019 and, respectively, 7692/13 May 2021). The following principles were respected: the principles of the Helsinki declaration of 1975, the European Convention of Oviedo of 4 April 1997 for the protection of human rights and the dignity of the human being in relation to the applications of biology and medicine. Prior to their inclusion in the study, all participants enrolled signed the informed consent and all obtained results were kept in an anonymous manner.

### 2.2. Subjects 

The control group included 40 subjects from the Integrated Outpatient Department of the Cluj-Napoca County Clinical Hospital of Infectious Diseases without thyroid pathology or other comorbidities. The case group included 40 patients with HT from the Cluj-Napoca County Clinical Hospital of Infectious Diseases with typical symptoms of hypothyroidism, 13 of them having comorbidities (8 with hypertension, 4 with type 2 diabetes, and 1 with a dermatological condition). Thyroid goiter was highlighted in 6 cases, while 7 patients had thyroid atrophy, and none of the patients with HT had an ophthalmological condition. The case group included patients diagnosed by endocrinologists, based on clinical criteria (fatigue and sluggishness, constipation, dry skin, increased sensitivity to cold, etc.) and paraclinical criteria all values for thyroid markers, such as thyroid stimulating hormone (TSH), free thyroxine (FT4), anti-thyroid peroxidase (ATPO), and anti-thyroglobulin antibodies (ATG), according to the European and national guidelines for hypothyroidism [21,22,23,24]. The control group included healthy persons, without clinical manifestations regarding thyroid diseases and with normal reference values for TSH, FT4, ATPO, and ATG. 

The matching between the groups was performed according to two criteria: the gender of the study participants (31 women and 9 men in each group) and the age of the study participants (in the control group the average age was 48.33 +/− 11.88 years, in the case group the average age was 47.35 +/− 11.18 years, the age range was 20–69 years in both groups).

### 2.3. Laboratory Investigations

#### 2.3.1. Thyroid Markers Analysis 

The paraclinical tests, as inclusion criteria for HT, targeted the following positive markers: TSH > 5.6 IU/mL, FT4 < 1.12 IU/mL, ATPO > 9 IU/mL, and ATG > 10 IU/mL, determined by the chemiluminescent immunoassay method, using the Unicel DXI 800 Analyzer (Beckman Coulter, Brea, CA, USA). Regarding immunological tests, blood samples collected in a sterile 6 mL vacutainer without anticoagulant were centrifuged for 10 min at 1800× *g* to obtain the serum. The markers of interest, such as TSH, FT4, ATPO, and ATG, were determined from the serum from each person enrolled in the study (case group and control group) according to the previously described method. 

#### 2.3.2. Molecular and Genetic Analyses

##### DNA Extraction

DNA was extracted in sterile 2 mL EthyleneDiamineTetraacetic Acid (EDTA) vacutainers using the ISOLATE II Genomic DNA Kit (Bioline, London, UK) based on special filter columns. 

Thus, for DNA extraction of each subject, 200 µL of blood were mixed with 200 µL of G3 lysis buffer and 25 µL of proteinase K and incubated at 70 °C for 10 min. For DNA precipitation, 200 µL absolute ethanol was added after incubation, and the resulting mixture was transferred to a column and centrifuged for 1 min at 10,000× *g*. In order to purify the DNA, several consecutive DNA washes were performed in sterile tubes, followed by centrifugation for 1 min at 10,000× *g*. The first wash was performed with buffer GW1 (reagent from the kit) and the second with buffer GW2 (reagent from the kit, reconstituted with absolute ethanol). After the last wash and the third centrifugation on the column, the DNA was eluted from the column transferred to a sterile tube in TRIS buffer (reagent from the kit) preheated (70 °C) in advance, and, finally, it was subjected to a final centrifugation for 1 min at 10,000× *g*. Immediately after extraction, the DNA was concentrated. In parallel, the purity of the extracted DNA was analyzed using the spectronanophotometer (NanoPhotometer P300, Implen GmbH, Munich, Germany). 

On average, a DNA concentration of 22 ng/µL with +/− 1.2 was obtained, with a purity at 260/280 nm of 1.7 +/− 0.17. All DNA samples were stored at −20 °C until genotyping.

##### Genotyping 

To prepare the Polymerase Chain Reaction (PCR) amplification, the following reagents were mixed to a final volume of 25 µL: in 12.5 µL Mastermix MyTaq™Red Mix (Bioline, London, UK), 9.5 µL of water-free of DNA/RNA-ase and 1 µL of each type of primer (forward and reverse primer), and, finally, 1 µL of genomic DNA were added. The fragment amplified with the PCR method had a length of 153 nucleotides. 

The following primers, 5′-AAGGCTCAGCTGAACCTGGT-3′ and 5′-CTGCTGAAACAAATGAAACCC-3′ (Ganeri Biotech, Hradec Králové, Czech Republic), were used, made according to available data [40], and PCR amplification was performed in a Biometra-TProfessional Basic Thermocycler (Analytik Jena GmbH, Jena, Germany). The initiation of the PCR reaction was carried out by activating the enzyme at 95 °C for 5 min, followed by 35 consecutive cycles that included DNA denaturation at 95 °C for 30 s, primer alignment at 54.5 °C for 30 s, extension/primer elongation at 72 °C for 30 s, and finally, after these steps, a final extension at 72 °C for 5 min. To verify the PCR reaction efficiency, agarose gel electrophoresis was performed using 10 μL ethidium bromide as a fluorochrome for 120 mL 2% gel. From each sample, 5 μL of the PCR products were loaded into the agarose gel. After that, the remaining 20 μL from the PCR products was purified using the ISOLATE II PCR Gel kit (Bioline, London, UK). Sample preparation was performed by adjusting the volume by adding 30 μL purified water. The final volume of 50 μL (PCR amplification and water) was mixed with 100 μL of Binding Buffer CB (reagent from the kit). The resulting mixture was transferred to a column and centrifuged for 30 s at 11,000× *g*. To purify the amplicons, several consecutive washes were performed in sterile tubes, followed by centrifugation for 30 s at 11,000× *g*. The wash was performed with buffer GW1 (reagent from the kit, reconstituted with absolute ethanol). After the second wash, the silica membrane was dried by centrifuge for 1 min at 11,000× *g* to remove residual ethanol. In the last step, 15 μL Elution Buffer C was added directly to the silica membrane, incubated at room temperature for 1 min, and centrifuged for 1 min at 11,000× *g*. As we mentioned before, DNA polymerase activity was checked by agarose gel electrophoresis, and the *CTLA-4* gene, compared to the molecular weight marker, was evidenced at 153 nucleotides. A negative control, DNA–RNA-free water, was used to verify the presence of contamination for the amplification process. Nucleotide incorporation errors produced by DNA polymerase due to the lack of a 3′–5′ exonuclease repair activity do not systematically occur at the same position in all amplified fragments. These differences will not be visible on in agarose gels electrophoresis and will not affect the molecular diagnostics. A polymorphism generated by nucleotide incorporation errors by DNA polymerase would be present in a proportion below the detection limit of the method. All PCR-purified products were used for Sanger sequencing. 

##### Sanger Sequencing

To sequence the samples, the purified PCR products (from each investigated subject) were stored in a pair of sterile tubes, as follows: 5 μL of a purified amplicon and 5 μL of the Forward primer were introduced into a sterile tube, and in the same way, 5 μL of the purified amplicon and 5 μL of the Reverse primer were introduced in another sterile tube, and after that the tubes were sealed and sent to Humanizing Genomics Macrogen Europe Company (Amsterdam, The Netherlands) which was performed the sequencing. The Sanger sequencing was performed in both directions. The obtained sequences were edited and analyzed using free, open-source bioinformatics software such as Geneious Prime 2021 (Biomatters Ltd., Auckland, New Zealand) and UGENE version 40.1 (Unipro, Novosibirsk, Russia). The new polymorphism associated with HT and highlighted by Sanger sequencing, which we found, cannot be a consequence caused by DNA polymerase errors.

### 2.4. Statistical Analysis 

For the statistical analysis, Pearson’s chi-squared test and Fisher’s exact test were applied with EpiInfo software, version 7.2.5.0. (CDC, Atlanta, GA, USA). The results were compared with those available in the large anthropological databases GenBank/BLAST, available via the National Center for Biotechnology Information (Bethesda, MA, USA). The results were considered to have statistical significance at the value of *p* < 0.05.

## 3. Results

Our interest was focused on the following thyroid markers: TSH, FT4, ATPO, and ATG. The results of thyroid markers analyzed for each subject were allowed inclusion in the HT group or in the control group, according to the mentioned criteria (into the Section 2.3.1). 

Table 1 shows the thyroid markers results found in the patients with HT.

The results of thyroid markers analyzed for each subject from the control group (without autoimmune thyroid disease) are presented in Table 2. 

The *CTLA-4* gene was successfully genotyped in all 40 patients enrolled in the study, as well as from the 40 subjects in the control group. The A22G and C55A polymorphisms were most frequently highlighted by Sanger sequencing. 

**Figure 1:**
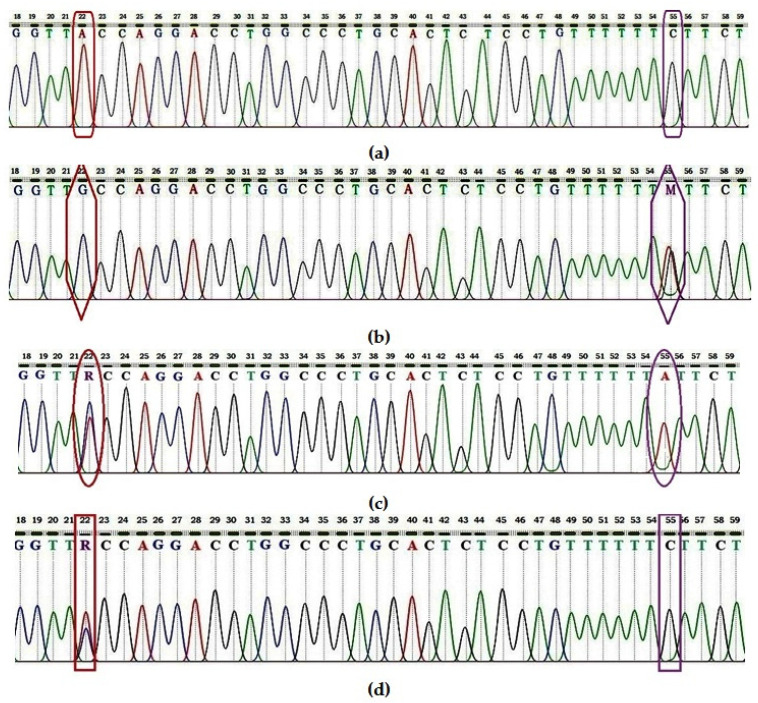
Chromatograms obtained by Sanger sequencing of the *CTLA-4* gene in subjects with Hashimoto’s thyroiditis compared to those in the control group: (**a**) The normal sequence of the gene in which the presence of A in position 22 (rs231775) and C in position 55 is observed. (**b**) The sequence with G in position 22 and M (heterozygous SNP with A and C) in position 55. (**c**) The sequence with R (heterozygous SNP with A and G) in position 22 and A in position 55. (**d**) The sequence with R (heterozygous SNP with A and G) in position 22 and C in position 55.

As can be seen in Figure 1, the polymorphisms of interest in the *CTLA-4* gene in exon 1 were A22G and C55A, located at 33 nucleotides from the first. 

Table 3 shows the genotyping results and distribution of *CTLA-4* alleles in exon 1 (position 22) found in the patients with HT, comparatively with the subjects from the control group, and summarizes the subsequent statistical analysis.

The frequency of the GG genotype in position 22 already described by others was not significant enough in patients with HT compared to the controls (15% vs. 7.5%, *p* = 0.51, OR 1.63, 95% CI 0.35–8.95).

Table 4 shows the genotyping results and distribution of *CTLA-4* alleles in exon 1 (position 55) found in the patients with HT, comparatively with the subjects from the con-trol group, and summarizes the related statistical analysis.

The frequency of the AA genotype at position 55 in HT patients was significantly higher, at 35, compared to the control group (35% vs. 0%, *p* < 0.05). Allele A in position 55 of the sequenced product was present in only one subject from the control group (hetero-zygous). The frequency of the A allele in patients with HT was much higher compared to healthy control patients (48.75% vs. 1.25%, *p* ≤ 0.05, OR 75.47, CI 95% 1.08–3.81).

We established for the first time that the genetic marker C55A is positively associated with HT.

## 4. Discussion

In this study, no statistically significant association was found between the polymorphism +49A/G (rs231775) position 22 in exon 1 of the *CTLA-4* gene and the genetic predisposition to HT. In contrast, a strong association between C55A in exon 1 of the *CTLA-4* gene and HT was highlighted. Testing the presence of the *CTLA-4* gene in other populations, the researchers did not find statistically significant associations between SNPs rs3087243 and autoimmune diseases such as HT (*p* > 0.05) [41,42]. The frequency of the A/G genotype and, respectively, of the G allele was not increased in the AITDs group. In contrast with the results of other studies conducted on populations from Europe and Asia [43,44] that claim that the A/A genotype seems to have a protective effect, our study did not confirm this hypothesis. Position 22 does not change significantly with age or sex, it was found that it is instead variable among different ethnic groups; the A22 allele is predominant in Europe, while the G22 allele is the more common in the Asian population [44,45]. Studies show that patients with A/G heterozygosity develop HT and Graves’ Disease (GD) more frequently, even if a great genetic variability is observed [43,44,45]. 

It was demonstrated in 2022 that the G22 allele is more prevalent among AITD patients [46]. The relationship between G22 and AITDs was constantly present in European and Asian people [44,45,46]. Our results did not demonstrate that the A/G genotype is interconnected with the development of HT. The CTLA-4 A49G genotypes in Polish patients confirmed that the CTLA-4 A/G genotype was significantly more frequent in the AITDs group, suggesting an increased susceptibility to HT. The same study from Poland revealed a statistically significant relationship with the presence of the G allele in GD patients, while in controls, the frequency of the CTLA-4 A/A genotype was significantly increased, suggesting a protective effect. This study found no statistically significant differences in the frequencies of other genotypes and polymorphic alleles of the CTLA-4 gene (1822 C/T and CT60 A/G) between the studied groups [14,15,31]. A significantly increased prevalence of the G/G genotype in Polish individuals diagnosed with HT was revealed [46,47]. These results are in line with many others centered on the *CTLA-4* gene rs231775 polymorphism in HT patients [44,48,49]. The results of another study in Polish children indicated that the CTLA-4 +49 GG genotype was significantly more frequent in both HT and GD patients, while the AA genotype was more frequent in controls [14,15]. The same study revealed that the CTLA-4 -318 CT genotype was significantly more frequent in AITDs, and the CC genotype occurred more often in controls [15]. Significantly higher median values of TPOAb and TgAb thyroid autoantibodies were associated with the G allele in HT and the T allele in GD patients [14,15]. It has been demonstrated, especially in adults, that A49G polymorphism in the *CTLA-4* gene was associated with an increased HT risk [16].

In our study, both the frequency of A and G alleles were approximately equal in the case group and the control group. Regarding GD, it should be emphasized that the *CTLA-4* gene is recognized as the most important genetic factor in the development of this pathology, from the perspective of the presence of the A22G point mutation in the *CTLA-4* gene [50]. In addition, it is established by researchers in the field that the G/G genotype in the *CTLA-4* gene, the A22G polymorphism is an important risk factor for HT-associated ophthalmopathy [50,51]. 

In contrast, in the present study, an association of the G/G genotype at position 22 with thyroid ophthalmopathy was not observed. In families with a history of AITDs, a preferential transmission of the G allele of the *CTLA-4* 22A > G SNP in exon 1 could be highlighted [15]. Similar results were obtained for European and Japanese patients with AITDs [52]. The *CTLA-4* G/G genotype of the A22G G > A SNP in position 22 is significantly increased according to existing studies [49,53,54]. The 22A > G SNP in the signal peptide of the CTLA-4 protein, which correlates the substitution of threonine with alanine, will cause less efficient glycosylation in the endoplasmic reticulum and reduced expression of the CTLA-4 surface protein [13,17,53,54]. This mutation results in increased activation of T lymphocytes by affecting the function and/or expression of the CTLA-4 protein [17,53,54,55,56,57]. The presence of the mutation increases the sensitivity of CTLA-4 inhibition receptors for thyroid cells [17,18,55]. 

Several studies compare the frequencies of *CTLA-4* gene alleles and genotypes into GD group with the HT group; high frequencies of G alleles and low frequencies of the A/A genotype were found in the group of patients with GD [37,38,39,40,41,42]. Based on the analysis of the A49G polymorphism in the *CTLA-4* gene, statistical analysis highlighted the fact that genotypes A/G and G/G increase the susceptibility to autoimmune pathology, such as GD [14]. Also, the *CTLA-4* gene is involved in mediating the suppressive functions of Treg (T cells regulation), in preventing autoimmunity, and its expression is also induced on activated T cells [27]. 

The same review presents the investigations of other SNPs in the *CTLA-4* gene, such as rs231775 (A49G), finding that carriers of the G allele have a higher risk of presenting AITDs (especially GD) than carriers of the A allele [17]. The author emphasizes the association between the rs231779 polymorphism and AITDs (including GD), with subjects with the T allele having a higher risk of hypothyroidism compared to those with the C allele [17]. SNPs (such as rs5742909) with no significant associations with AITDs were also reviewed [17]. 

However, the phenotype in autoimmune thyroid diseases is not determined only by *CTLA-4* polymorphisms [17,48]. Nevertheless, studies contradict each other [14,42,56,57]. Thus, some authors suggest that some persons with the G/G genotype (rs 231775) in the *CTLA-4* gene will not develop autoimmune thyroid diseases, while those with the A/A genotype may develop AITDs [14,17,42]. Lack of an association between SNP 49A/G (rs231775) and HT, as the authors of other studies found, was partly due to the small size of both groups, and possibly to population characteristics. Other limitations were given as the size of the groups studied and the used method for sequencing, even if it is considered the “gold standard” for SNPs analysis. We selected the Sanger sequencing method, which, even if it is laborious and time-consuming, lends itself to being used for genotyping a small group of samples, thus respecting an optimal cost-effectiveness ratio. 

No association between this C55A SNP and genetic predisposition to HT has been reported in the literature at the time of writing this article. The only mention of this 55/C > A mutation was reported by the Invitae Corp. (San Francisco, CA, USA). The study exclusively presented the mutation, without demonstrating the association with certain autoimmune diseases [58]. 

Here, in our study, we report for the first time in the literature the positive association between the 55/C > A polymorphism and the development of HT. The presence of this point mutation in position 55/C > A will result in the substitution of leucine with isoleucine, which could determine the reduction in the expression of the CTLA-4 surface protein. In our opinion, there is a multifactorial etiopathogenesis of HT and GD, which could also be closely related to the polymorphisms present at the major histocompatibility complex (MHC) class II alleles, which would open the way for other studies.

## 5. Conclusions

In this study we found that patients with the GG genotype of the *CTLA-4* exon 1 A22G gene polymorphism did not show increased susceptibility for developing Hashimoto’s thyroiditis.

The presence of the point mutation in position 55/C > A, which results in the substitution of leucine with isoleucine instead, observed for the first time in our study in association with Hashimoto’s thyroiditis, determines a low expression of the CTLA-4 surface protein. To confirm this hypostasis, further studies are needed. It was demonstrated in our study that the A allele (position 55, on the amplified exon 1 of the *CTLA-4* gene) is associated with the presence of the disease, being almost absent in the control group. Consequently, the results of our study demonstrated, for the first time, a genetic relationship between the *CTLA-4* (+55A/GG) genotype and the seropositivity of the thyroid autoantigens TPO and TG. To date, there is no other evidence available regarding this point mutation and its phenotypic effect in Romanian patients with AITDs and other parts of the world. 

## Figures and Tables

**Table 1:** The thyroid markers values in the group of patients with Hashimoto’s thyroiditis (HT).

Subject Number	Sex	Age	TSH Values (IU/mL)	FT4 Values (IU/mL)	ATPO Values (IU/mL)	ATG Values (IU/mL)
1	M	44	0.73	1.50	15.10	<1
2	F	53	0.67	1.51	755.00	<1
3	F	69	2.95	0.87	7.52	560.57
4	F	44	3.25	1.10	16.30	17.00
5	F	51	5.16	0.72	1342.30	967.20
6	M	46	1.43	0.85	577.80	<1
7	F	58	0.59	0.84	201.50	<1
8	F	57	1.04	1.29	34.10	224.90
9	F	56	1.31	0.76	305.50	3.11
10	F	54	7.10	1.24	463.40	360.50
11	F	29	21.00	0.10	200.00	126.53
12	F	32	1.02	0.10	15.40	56.00
13	F	44	1.02	0.10	29.00	47.00
14	F	35	1.56	14.97	10.10	11.00
15	F	57	4.76	0.72	307.40	1.00
16	M	42	2.10	12.10	8.14	14.60
17	F	41	3.10	8.90	8.14	14.00
18	M	43	19.20	0.67	4056	316.20
19	F	35	4.50	0.10	883.00	0.60
20	F	20	3.60	0.20	1081.00	10.00
21	M	53	10.07	0.66	1014.00	31.50
22	F	50	178.00	1.04	1192.00	0.60
23	M	52	8.54	0.53	1081.00	9.70
24	F	43	0.72	0.70	140.70	2.00
25	F	45	5.82	0.64	128.90	0.60
26	F	57	38.98	0.53	5.00	0.80
27	M	57	2.12	0.82	342.00	1.60
28	F	49	1.23	0.80	3.60	2.20
29	F	68	5.14	0.91	83.20	2.30
30	F	42	5.66	0.95	179.00	389.20
31	F	43	0.04	0.59	8.10	569.20
32	F	42	0.01	1.18	119.10	3.20
33	F	61	1.35	0.64	0.80	18.20
34	F	53	0.36	1.19	740.10	10.60
35	F	45	3.42	0.91	411.20	67.00
36	M	63	8.17	0.52	213.90	166.40
37	M	27	5.02	0.23	1041.00	450.00
38	F	44	7.50	0.52	16.30	14.00
39	F	61	7.50	0.52	16.30	14.00
40	F	29	0.09	0.47	19.10	27.10

TSH: thyroid stimulating hormone; FT4: free thyroxine; ATPO: anti-thyroid peroxidase; ATG: anti-thyroglobulin antibodies.

**Table 2:** The thyroid markers values in the control group (without autoimmune thyroid disease).

Subject Number	Sex	Age	TSH Values (IU/mL)	FT4 Values (IU/mL)	ATPO Values (IU/mL)	ATG Values (IU/mL)
1	F	60	3.51	0.69	7.90	3.08
2	F	69	4.80	0.70	6.80	1.92
3	F	45	0.95	0.97	5.70	2.34
4	F	53	1.72	0.98	1.80	2.69
5	F	35	1.06	0.66	3.66	3.50
6	M	52	2.66	0.69	5.86	2.21
7	F	41	1.07	0.78	2.16	3.20
8	M	63	0.83	0.96	5.46	1.66
9	F	44	5.02	0.91	3.98	1.83
10	F	43	3.20	0.88	2.10	1.92
11	M	57	4.16	0.85	6.90	1.79
12	F	54	3.22	0.96	4.11	2.68
13	F	58	2.10	0.99	2.34	1.99
14	F	29	3.11	0.71	4.35	2.11
15	F	61	4.80	0.76	3.49	2.95
16	M	53	1.20	0.68	4.99	3.20
17	F	45	2.17	0.83	5.10	1.72
18	F	53	2.98	0.89	3.80	0.82
19	F	32	0.48	1.10	1.40	2.30
20	M	46	0.87	0.81	2.00	3.16
21	F	60	1.40	0.62	3.00	3.40
22	F	68	1.80	0.68	4.60	0.95
23	F	29	2.30	0.90	3.70	1.78
24	F	65	3.20	0.91	2.50	2.90
25	F	44	4.70	0.71	3.60	2.67
26	F	42	1.60	0.74	2.60	2.45
27	M	44	1.20	0.82	2.56	3.10
28	F	44	3.60	0.99	2.39	3.01
29	F	51	4.10	0.91	1.40	1.56
30	M	43	5.10	0.63	2.40	2.02
31	F	61	2.60	0.70	3.40	2.72
32	F	50	1.90	0.65	7.10	1.69
33	F	42	2.50	0.90	8.90	1.29
34	M	27	2.60	0.76	2.67	1.73
35	F	30	5.12	0.81	5.61	2.29
36	F	20	4.18	0.90	6.62	2.49
37	F	58	3.06	0.94	<1	<1
38	F	55	2.10	0.90	<1	<1
39	M	58	3.15	0.93	4.56	2.19
40	F	49	2.01	0.80	3.67	1.75

TSH: thyroid stimulating hormone; FT4: free thyroxine; ATPO: anti-thyroid peroxidase; ATG: anti-thyroglobulin antibodies.

**Table 3:** Distribution of *CTLA-4* gene polymorphisms in exon 1 position 22, in the group of patients with Hashimoto’s thyroiditis (HT), compared to the control group.

	Hashimoto Group, *n* = 40, Position 22 (%)	Control Group, *n* = 40 (%)	OR	95% CI	*p*-Values
Genotype *					
AA	23 (57.3)	19 (47.5)	1.48	0.61–3.65	0.37
AG	11(27.5)	18 (45)	0.5	0.18–1.34	0.16
GG	6 (15)	3 (7.5)	1.6	0.35–8.95	0.51
Allele frequency					
A	57	56	1	0.53–2.11	0.86
G	23	24	1	0.47–1.87	0.86

* The fragment amplified by the PCR method has a length of 153 nucleotides, and position 22 suggests the change of nucleotide A with G.

**Table 4:** Distribution of *CTLA-4* gene polymorphisms in exon 1 position 55, in the group of patients with Hashimoto’s thyroiditis (HT), compared to the control group.

	Hashimoto Group, *n* = 40, Position 55 (%)	Control Group, *n* = 40 (%)	OR	95% CI	*p*-Values
Genotype *					
CC	15 (37.5)	39 (97.5)	27.11	4.12–623.67	<0.05
AC	11 (27.5)	1 (2.5)	-	-	<0.05
AA	14 (35)	0 (0)	-	-	-
Allele frequency					
A	39 (48.75)	1 (1.25)	75.47	13.37–1555.76	<0.05
C	41 (51.25)	79 (98.75)	-	-	-

* The fragment amplified by the PCR method has a length of 153 nucleotides, and position 55 suggests the change of nucleotide C with A.

## Data Availability

The data included in this paper are available from the corresponding author. Data are not publicly available due to patient privacy.
